# Hierarchical Bayesian non-response models for error rates in forensic black-box studies

**DOI:** 10.1098/rsta.2022.0157

**Published:** 2023-05-15

**Authors:** Kori Khan, Alicia Carriquiry

**Affiliations:** ^1^ Department of Statistics, Iowa State University, Ames, IA, USA; ^2^ Center for Statistics and Applications in Forensic Science, Ames, IA, USA

**Keywords:** black-box study, Bayesian inference, forensic science, item non-response, non-ignorable missingness

## Abstract

Forensic science plays a critical role in the United States criminal legal system. Historically, however, most feature-based fields of forensic science, including firearms examination and latent print analysis, have not been shown to be scientifically valid. Recently, black-box studies have been proposed as a means of assessing whether these feature-based disciplines are valid, at least in terms of accuracy, reproducibility and repeatability. In these studies, forensic examiners frequently either do not respond to every test item or select an answer equivalent to ‘don’t know’. Current black-box studies do not account for these high levels of missingness in statistical analyses. Unfortunately, the authors of black-box studies typically do not share the data necessary to meaningfully adjust estimates for the high proportion of missing responses. Borrowing from work in the context of small area estimation, we propose the use of hierarchical Bayesian models that do not require auxiliary data to adjust for non-response. Using these models, we offer the first formal exploration of the impact that missingness is playing in error rate estimations reported in black-box studies. We show that error rates currently reported as low as 0.4% could actually be at least 8.4% in models accounting for non-response where inconclusive decisions are counted as correct, and over 28% when inconclusives are counted as missing responses. These proposed models are not the answer to the missingness problem in black-box studies. But with the release of auxiliary information, they can be the foundation for new methodologies to adjust for missingness in error rate estimations.

This article is part of the theme issue ‘Bayesian inference: challenges, perspectives, and prospects’.

**With gratitude, to Sir Adrian F. M. Smith**

Adrian Smith is a giant in statistics, so adding our work to this volume in Adrian’s honour is a huge privilege. Much has been said about Adrian’s extraordinary, game-changing contributions to statistics, especially to Bayesian statistics. What may be less well known is that Adrian is also a genuinely nice guy, with a warm heart and a wicked sense of humour. He made a profound and lasting impression on many of us who had the good fortune to interact with him early in our professional journey. We are forever grateful!

## 1. Introduction

The United States has the highest rate of incarceration among developed countries. According to official national statistics, about 630 per 100 000 persons were behind bars in the United States in 2021. By contrast, the proportions were 214, 130 and 70 per 100 000 in Australia, England and Germany during the same period, respectively. The reasons behind mass incarceration in the US are many and complex, and beyond the scope of this work. One salient fact, however, deserves mention. Incarceration at this scale is bound to result in a high number of wrongfully convicted persons. While the exact number is unknowable, innocence organizations estimate that there are between 20 000 and 200 000 persons in prison today for crimes they did not commit. In the last three decades, over 3200 innocent persons were exonerated [1], but not everyone has been so fortunate. Several wrongfully convicted individuals have been executed before their innocence could be established [2]. A leading cause of wrongful convictions is the use of *ad hoc*, subjective methods to evaluate evidence, and the exaggerated claims by expert witnesses during trial (e.g. [2]).

**Figure 1.**
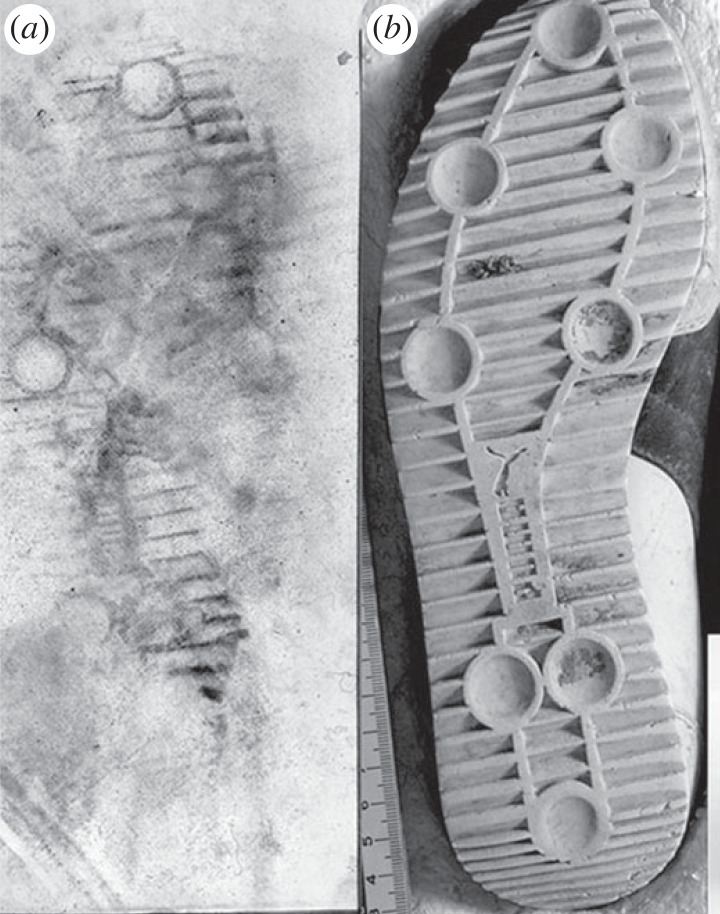
(*a*) Footwear print left at a crime scene and (*b*) photograph of putative shoe.

Highly publicized failures in the application of forensic technologies including microscopic hair comparisons, the analysis of bullet lead and forensic bitemark analysis in the early part of the century [3] led to the National Academy of Sciences, Engineering and Medicine (NASEM) in the United States to charge a panel of experts with the review of the scientific underpinnings (if any) of most forensic disciplines. The panel issued a report (henceforth ‘NAS’ report) entitled *Strengthening Forensic Sciences in the United States: A Path Forward* [4] that was sharply critical of many forensic disciplines. Single-donor forensic DNA analysis was highlighted as the gold standard discipline for individualization. The panel was particularly disparaging of what is known as feature or pattern comparison disciplines, including latent print analysis, footwear and tire tread analysis, firearms and toolmark examinations, among others. In these disciplines, the evidence recovered from a crime scene is often an image that is then compared to other images obtained from a suspect or from other crime scenes. For illustration, figure 1 shows a shoe print that was left by the perpetrator of a crime (figure 1*a*) and the image of the outsole of the suspect’s shoe (figure 1*b*). The question of interest is whether the images are ‘similar enough’ to place the suspect at the crime scene.

Examiners evaluating this type of evidence rely on visual comparison of the images, perhaps aided by tools such as Photoshop and a magnifying instrument. At this stage, examiners may decide that the images are unsuitable for comparison. If the images are suitable for comparison, examiners then report the results of the evaluation using a categorical scale that may include three possible conclusions—identification, exclusion or inconclusive—as in the case of US firearms examinations, or up to nine gradated conclusions as in the case of US questioned document examiners. Because of the absence of measurements or other quantitative criteria underpinning their conclusions, standard metrics to assess accuracy and reliability of examiners are not available.

As a follow-up to the NAS report, in 2016, the President’s Council of Advisors in Science and Technology (PCAST (2016)) focused on pattern comparison disciplines and reported that their *foundational validity* still needed to be established. To do so, PCAST (2016) recommended the use of black-box studies. Since then, black-box studies have been conducted in multiple forensic disciplines (see e.g. [5–12], appendix A(a) organizes recent black-box studies by discipline). The results often suggest that examiners tend to be remarkably accurate, hardly ever committing an error. For example, a recently completed study with firearms examiners resulted in less than 1% false positive rate. These seemingly low error rates have been presented in court as proof that forensic examiners rarely, if ever, make a mistake.

However, a closer look at the design and analysis of several recent black-box studies paints a different picture. Self-selected participants, high rates of attrition, item non-response that is likely to be non-ignorable, and the way in which inconclusive responses are used when computing error rates instead suggest that examiners’ accuracy is likely to be much lower than what has been published [13]. Reproducibility (inter-examiner consistency) and repeatability (intra-examiner consistency) tend to be low, another indication that the probability of erroneous conclusions by examiners has been underestimated in many cases.

In this work, we focus on missingness in black-box studies and its impact on estimates of accuracy or error rates. We propose modelling approaches to estimate error rates that account for the missing responses. The paper is organized as follows: In §2, we provide a brief background of black-box studies, highlighting both the experimental design of these studies and the existing rates of missingness. In §3, we introduce types of missingness mechanisms. Focusing on black-box studies, we provide an overview of how a Bayesian approach for non-response could work and explore the current barriers to developing suitable models. We then define a set of Bayesian hierarchical models in §4. These models, which include one for ignorable and one for non-ignorable missingness, require no auxilary information to be fit. We show how they can be used to approximate the posterior predictive distribution of a random variable that represents the expected proportion of errors in a study. We then use these models to re-analyse the data from a recent black-box study of latent palmar prints in §5. We conclude in §6 with a discussion of how our proposed models can be used by the forensic and legal communities and some directions for future work.

## 2. Black-box studies

In this section, we provide a background on the origins of black-box studies and typical features of their experimental designs. We also provide an overview of what is currently known about missingness in black-box studies.

### (a) Design of black-box studies

The PCAST (2016) report stated that empirical studies must show that a forensic method is accurate, repeatable and reproducible [14]. However, forensic feature comparison disciplines are difficult to evaluate empirically. This is, in part, due to the fact that they rely on subjective decisions. In response to this complication, PCAST (2016) suggested using black-box studies to assess the scientific validity of these disciplines.

In these studies, researchers compile test sets or items for comparison. Each item ideally consists of one comparison between a sample of unknown origin (e.g. a latent print) and a sample of known origin (e.g. one or more latent prints taken from a known individual). These samples will either be from the same source or a different source. To ensure error rates can be calculated, the researchers know the ground truth. However, the examiners do not. The examiner is then asked to decide whether the samples came from the same source.

Using the known ground truth for these items, researchers then arrive at measures of accuracy, repeatability and reproducibility. Accuracy is measured by the rate at which examiners obtain correct results both for same source and different source items. Repeatability is measured by the rate at which the same examiner arrives at a consistent conclusion when re-examining the same item. Reproducibility is the rate at which two different examiners reach the same conclusion when evaluating the same sample.

Importantly, item comparisons in black-box studies are almost never pair-wise independent of one another. By design, each participating examiner will typically render multiple comparison decisions in a given study. For studies measuring repeatability, not only will a single examiner analyse multiple items, he/she will also analyse the same items repeatably. Additionally, in all black-box studies we have observed, the same items are compared by multiple examiners. For example, in a latent palmar print black-box study considered in §5 each item with a comparison decision was analysed by an average of 23 examiners [5].

In the vast majority of black-box studies, researchers also collect information about the participating examiners and/or the characteristics of the items assigned to examiners. Most commonly, this information is in the form of a background survey about the examiners’ qualifications or training. However, more recent black-box studies have also gathered information about the quality of the items themselves.

For the rest of this paper, unless otherwise specified, we focus on accuracy measures. More specifically, we focus on two common accuracy measures of interest in black-box studies: the false positive rate and the false negative rate. The false positive rate is the proportion of times examiners erroneously conclude that two samples came from the same source; while the false negative rate is the proportion of times examiners erroneously conclude that two samples came from different sources.

### (b) Missingness in black-box studies

Current black-box studies are plagued by missingness problems. This missingness occurs both for the response (comparison decisions on items) and potential covariates (examiner or item characteristics). However, at the time of writing, there are no studies that release sufficient information about the covariates to explore this concern, and so we focus on missingness in the response variable in this paper.

There have been no formal attempts to account for missingness in error rate estimations, and this paper marks the first effort to begin to tease apart the different types of missingness that are typically present in black-box studies. We start by distinguishing between unit and item non-response (see e.g. [15]). For us, unit non-response in the response occurs when an examiner agrees to participate in a study but fails to respond to even one comparison item. Item non-response in the response occurs when an examiner responds to at least one comparison item, but not all of the assigned items. The unit and item non-response rates may vary across analyses. For example, it is possible, and even likely, that both the item and unit non-response rates may differ for different source and same source items. However, at the time of the writing, there are only two black-box studies that have released sufficient information to calculate the unit and item non-response rates for any individual analyses conducted [5,8]. One of these, by Eldridge *et al.* [5], is considered in §5 and we explore examples of the non-response rates for it in the next paragraph.

When considering unit non-response across all possible analyses, it is common to see rates over 20% in black-box studies [5,10,11,16–18]. Similarly, when it is calculable, item non-response calculated in this manner is also over 20% in multiple studies [5,17]. For the black-box studies that have released information, both the unit non-response and item non-response rates for a given analysis can be even higher. For example, in the black-box study of latent print examiners considered in §5, the unit non-response rate was 44% and the item non-response rate was 37% for the false positive error rate calculations.

In the above discussion, the response rates typically treat any response to an item as being an observed response—even if an examiner did not actually arrive at a definitive comparison decision. One of the complications of black-box studies is defining what exactly it means to be missing. The process of arriving at a comparison decision typically involves several steps. While these steps can vary across disciplines, they look something like the following. First, the examiner is asked to assess the quality of the sample of unknown origin. Many disciplines have a categorical outcome for this stage. For example, latent print examiners often use three categories: value for individualization, value for exclusion only or no value [5,18]. Examiners will only proceed to the next step if the item is deemed suitable for comparison; if so, the examiner then arrives at another categorical conclusion about the origin of the unknown sample. The categories of this conclusion typically include: Identification (the samples are from the same source), Exclusion (the samples are from different sources) or Inconclusive (with different disciplines having various special cases of inconclusive). In practice, black-box studies simply drop items deemed of no value (or its equivalent) from the study. Additionally, almost all black-box studies count inconclusives as ‘correct’ decisions. This is known to be problematic [19,20]. Hofmann *et al.* [20] found that inconclusives were used at a much higher rate for different source items than same source items for firearms examiners, while Dorfman & Valliant [19] explored how the presence of inconclusives can negatively influence error rate estimates. However, treating inconclusives as correct remains the prevalent practice.

Practically speaking, a determination of ‘no value’ or a conclusion of inconclusive both result in a missing comparison decision for error rate calculations. The methodologies currently in use to estimate error rates rely on a binary response. Unfortunately, they can account for a large portion of the items with a response. For example, in the latent palmar print black-box study considered in §5, the authors reported that approximately 35% (4246) of the 12 279 items examiners responded to were deemed to be of no value or recorded as an inconclusive decision. If there were agreement among examiners about which prints are of no value (i.e. no one would compare such items) or which items (comparisons) were inconclusive, it might be reasonable to simply drop these items from an analysis designed to estimate error rates. However, approximately 33% of items marked as ‘no value’ by one examiner were found to be of value by other examiners (see fig. 3 in [5]). Similarly, the authors reported that examiners arrived at a decision of inconclusive for 19% (1840 items) of the 9460 items with a comparison decision rendered (see fig. 5 in [5]). Here, however, 61% of the time an examiner marked an item as ‘inconclusive’, another examiner was able to arrive at a comparison decision on the same item.

Intuitively, a response that an item is of no value for comparisons or a conclusion of inconclusive indicates that the examiner is unsure whether the item of unknown origin is from the same source or a different source from the samples of known origin. In other words, these responses are both variants of ‘don’t know.’ In the broader statistical literature, one prevalent view is that the best solution to ‘don’t know’ responses is to design experiments or surveys to discourage such responses [21]. However, this is by no means a consensus view, and the forensic science community feels strongly that examiners should be able to provide either answer. One option, however, might be to consider these values as missing responses in models accounting for non-response (see e.g. [22]). We note that doing so will greatly inflate the non-response rates. Returning to the black-box study of latent print examiners, treating inconclusives as missing leads to a unit non-response rate of 46% (compared to 44%) and an item non-response rate of 46% (compared to 37%) for the false positive rate calculations we previously considered.

## 3. Adjusting for missingness

There are many ways to adjust for missingness. However, choosing the appropriate method depends on the mechanism that lead to the missingness. In this section, we provide a brief overview of missingness mechanisms and existing Bayesian methods for adjusting for each with a focus on methods most relevant to error rate estimation in black-box studies. We also highlight the current barriers to developing more sophisticated methods for error rate estimation in black-box studies.

### (a) Missingness mechanisms

Rubin [23] was the first to formalize missing data mechanisms, but modern nomenclature has evolved [24,25]. Currently, missing data mechanisms are described as falling into one of three categories: missing completely at random (MCAR), missing at random (MAR) and not missing at random (NMAR) [24,25].

In the following subsection, we discuss each type in the context of black-box studies. For simplicity, we assume that every examiner is assigned the same number of item comparisons. As noted in §2, there are often both missing response variables and potential covariate variables. Currently, too few studies have released enough information about potential covariates to meaningfully incorporate them into a model. However, we introduce covariates into our discussion of missingness mechanisms here because we will discuss their potential incorporation into future non-response methods.

We define Y to be the I×J matrix of response data, where $y_{ij}$ is 0 if the ith examiner’s comparison decision on item j was correct and 1 otherwise. We define X to be the I×p matrix of covariates related to the examiners, where the ith row corresponds to the ith examiner. We will consider the matrix D to be the I×(J+p) combination of responses and examiner demographics (i.e. D=[Y X]). Finally, we define R be the I×(J+p) matrix where $r_{ij}$ is 1 when $D_{ij}$ is observed and 0 otherwise. We formalize missingness mechanisms by using probability distributions, and we assume that the missingness mechanism can depend on a vector of unknown parameters ϕ.

In the best case scenario, whether data are missing should not depend on any element of Y or X. More precisely, the following would hold:

3.1 f(R|Y,X,ϕ)≡f(R|ϕ) ∀ Y,X,ϕ.

In this case, the missingness would be MCAR. If, instead, the missingness depended only on observed values of the data matrix Y or X, i.e.:

3.2 $$f(R|Y,X,\phi )\equiv f(R|Y^{\mathrm{obs}},X^{\mathrm{obs}},\phi )\;\forall \;Y,X,\phi ,$$

then we would call it MAR. When the data missingness is either MCAR or MAR, it is often called ‘ignorable’. This is because in either case, point estimates are typically unbiased if the missingness is ‘ignored’. The most problematic missingness mechanism is NMAR. This occurs when f(R|Y,X,ϕ) cannot be simplified to the right-hand sides of either (3.1) or (3.2). NMAR missingness is often referred to as non-ignorable because analyses that fail to account for it will usually result in biased estimates. We adopt this nomenclature in this paper.

Note that the missingness for the response and the covariate variables need not be of the same type. For example, it is possible for the missingness in Y to be non-ignorable while it is MAR for X. We also note that we could have included another matrix to incorporate covariate information about the items themselves. The extension to this scenario would be straightforward and is omitted for simplicity.

Despite the high rates of missingness in the black-box studies, we are unaware of any efforts to explore the patterns of missingness or the impact that it may have on error rate estimates besides our own recent attempt. In Khan & Carriquiry [13], we explored the patterns of item non-response in the palmar print study described in §5, and we found evidence that there was non-ignorable item non-response.

### (b) Bayesian approaches to adjusting for missingness

There are a variety of statistical methods to deal with missing data. These can include maximum likelihood, multiple imputation and fully Bayesian (which we refer to as Bayesian) methods. Each approach has its own advantages and disadvantages. There have been numerous efforts to compare and contrast them (see e.g. [26,27]). In this section, we offer a brief overview of relevant Bayesian approaches. We do so with the focused goal of illustrating how well a Bayesian approach matches the data structure, inferential goals and current problems with statistical analyses in black-box studies. We then highlight current barriers to implementing these approaches. Readers interested in a more comprehensive overview of Bayesian methods for missing data may refer to Ma & Chen [28].

Broadly speaking, Bayesian methods treat missing data as random variables. Priors can be specified on the parameters defining the distribution of these variables, and Markov chain Monte Carlo (MCMC) methods can be used to draw samples from the relevant posterior distributions. This approach often requires parametric assumptions to be made about the missing data, which can be limiting [29]. However, it also allows for the simultaneous estimation of the missing data and the parameters, and the ability to account for the uncertainty the missing data contributes to the estimation of parameters.

In this paper, we use an approach often referred to as the selection model (SM) approach [25]. In this approach, the full data model is factorized as

3.3 f(Y,X,R|α,β,ϕ)=f(Y|X,α)f(X|β)f(R|Y,X,ϕ),

where α is the set of parameters governing the conditional distribution Y|X, β is the set of parameters governing the conditional distribution X|β and ϕ is defined as in §3(a). This approach requires specifying the response model, missing covariate distribution and missingness model, respectively.

In the context of black-box studies, there are immense advantages to the Bayesian method generally, and the SM approach in particular. The SM’s modular approach allows for the response model to be specified without a direct dependence on the missingness model. This has a practical appeal because the forensic science and legal communities are primarily interested in the response. Perhaps more importantly, in the Bayesian context, the modular approach allows for the opportunity to leverage dependencies in the data when adjusting for missingness at both the unit and item level in a conceptually simple way. As described in §2, each examiner responds to multiple items and often multiple examiners analyse each item. When an examiner omits a response to a given item, auxiliary information about the examiner and the item can be used in the response model. In the case that an examiner does not respond to a single item and auxiliary information is known about him/her, this will allow for potentially meaningful predictions of his/her responses.

In the context of black-box studies, a straightforward extension of the SM approach could include modelling the probability that an inconclusive (or decision that a mark was of no value) would have been an error. This could be incorporated into a hierarchical response model, thereby extending error rate estimations beyond binary responses. This approach could allow for a more formal exploration of the impact that inconclusives have on error rate estimations.

### (c) Barriers to adjusting for missingness in black-box studies

There are two related obstacles to developing relevant Bayesian models for error rate estimations in black-box studies. One is the lack of publicly available data. The PCAST (2016) report calls for public release of de-identified data from black-box studies. However, at the time of writing, only two black-box studies have released any portion of data for analysis by independent researchers. The first study [5], considered in §5, released information about all items examiners responded to as well as auxiliary information about both the items and the examiners. Importantly, they released no information about the items with no response. From a missing data perspective, this eliminates the ability to leverage information known about a particular item when modelling the response in an SM approach. The second study released aggregated information about participant performance and demographic information [8]. However, there is no link between an examiner and the demographic information, again hindering the ability to use the data for missing data models.

The second problem is that very little is known about feature-based comparison methods or the individuals who carry them out. The standards for serving as an expert witness for forensic analyses in a US trial are not high. For example, 2 days (or less) of formal training in a discipline can be sufficient to qualify as an expert in a forensic science discipline (see e.g. [30]). Few states have established regulatory boards to establish minimum requirements to be an examiner. This, unfortunately, means that it is very difficult to even identify the population of examiners likely to provide testimony in court proceedings. As a consequence, the scientific community knows very little about what makes a forensic examiner more (or less) accurate. Similarly, prior to black-box studies, there had been no meaningful empirical studies in feature-based comparison methods.

The combination of the lack of available data and lack of a broader understanding of factors related to the accuracy of forensic methodologies makes it practically difficult to develop methodology to adjust for missingness. For a Bayesian SM approach, this is particularly true as assumptions must be made to ensure parameters are identifiable and these assumptions could dominate inference if there is no data or intuition to guide choosing them [31].

It is in light of these restrictions that we propose a set of models that use no auxiliary information in the next section. Although our previous work has suggested that there is non-ignorable item non-response in at least one black-box study, many researchers recommend conducting sensitivity analyses to assess the potential impacts of different types of mechanisms when in doubt about the mechanisms [32]. In the rest of the paper, we make no assumptions about the missingness mechanism that led to the missingness in the data we analyse.

## 4. Hierarchical Bayesian models for missingness in black-box studies

In this section, we borrow from the small area estimation literature to propose a hierarchical Bayesian model for both ignorable and non-ignorable missingness. We also define an analogous model for completely observed data for comparison purposes.

Stasny proposed a set of hierarchical models to model non-response and the prevalence of a characteristic of interest in the context of small area estimation problems [33]. She proposed a model for both ignorable and non-ignorable missingness. She originally employed an empirical Bayes approach to fitting these models, but Nandram & Choi [34] subsequently proposed a fully Bayesian approach (see also [35]).

Although the context and inferential goals were different in these papers, the models proposed can be useful in the context of black-box studies. This is particularly true given the current lack of data available from black-box studies because these models only depend on observed error rates and non-response rates. Before we introduce the ignorable and non-ignorable models, we introduce a similar model intended for completely observed data. We do so because we want to give some insight into the impact of adjusting for missingness instead of ignoring it.

For all the following models, we let Y be the collection of $y_{ij}$ for $j=1,\ldots ,J_{i}$ and i=1,…,I, where $y_{ij}$ is an indicator of whether the ith examiner made an error on the jth item. Similarly, we define R to be the collection of $r_{ij}$ for $j=1,\ldots ,J_{i}$ and i=1,…,I, where $r_{ij}$ is an indicator of whether the ith examiner responded to the jth item. We let $R_{i}=\sum_{j=1}^{J_{i}}r_{ij}$ be the number of observed responses for examiner i. To simplify notation down the line, we assume that for fixed i, the $y_{i}=\{y_{i1},\ldots ,y_{iJ_{i}}\}$ are ordered such that the first $R_{i}$ observations are observed and the last $J_{i}-R_{i}$ observations are missing. Finally, we define $I^{*}=\sum_{i=1}^{I}I_{R_{i}>0}$ to be the number of examiners with at least one non-missing response.

Currently, black-box studies simply limit analyses to $y_{ij}$ that are observed (i.e. for which the corresponding $r_{ij}=1$). We would ultimately like to compare such an approach to approaches which adjust for missingness. Because most methods used to estimate error rates are frequentist, we introduce a Bayesian method here that can be used to analyse these observed data. We emphasize this model is for comparison purposes only. For $y_{ij}$; $j=1,\ldots ,R_{i}$ and $i=1,\ldots ,I^{*}$ we define the ‘naive’ model as follows:

**Model 4.1 (Naive).**

$$y_{ij}|p_{i}\overset{i.i.d.}{∼}\text{Bernoulli }(p_{i})$$

and

$$p_{i}|\mu_{p},\tau_{p}\overset{i.i.d.}{∼}\text{Beta}(\mu_{p}\tau_{p},(1-\mu_{p})\tau_{p}).$$

Here, $p_{i}$ is the probability that the ith examiner makes an error. This model allows the error rate to vary by examiner, as advocated for in several firearm black-box studies (e.g. [17]). However, it assumes that for a given examiner, the occurrence of errors is conditionally independent given the $p_{i}$.

We now turn to the ignorable model defined for $y_{ij}$ and $r_{ij}$; $j=1,\ldots ,J_{i}$ and i=1,…,I as follows:

**Model 4.2 (Ignorable missingness).**

$$\begin{array}{rl} & y_{ij}|p_{i}\overset{i.i.d.}{∼}\text{Bernoulli }(p_{i})\\ & r_{ij}|\pi_{i}\overset{i.i.d.}{∼}\text{Bernoulli }(\pi_{i}),\\ & p_{i}|\mu_{p},\tau_{p}\overset{i.i.d.}{∼}\text{Beta}(\mu_{p}\tau_{p},(1-\mu_{p})\tau_{p})\\ \mathrm{and}\;\; & \pi_{i}|\mu_{\pi },\tau_{\pi }\overset{i.i.d.}{∼}\text{Beta}(\mu_{\pi }\tau_{\pi },(1-\mu_{\pi })\tau_{\pi }). \end{array}$$

The $p_{i}$ are defined as before. Here, $\pi_{i}$ is the probability that the ith examiner responds to an item. This model allows the probability of response to vary by examiner. However, responses are assumed to be conditionally independent given $\pi_{i}$. Importantly, the probability of response does not depend on whether an error occurred.

Finally, the non-ignorable model is a straightforward extension of this model. Now, we allow the probability of response $\pi_{i}$ to differ depending on whether an error occurred. For $y_{ij}$ and $r_{ij}$ for $j=1,\ldots ,J_{i}$ and i=1,…,I, we define the non-ignorable model as follows:

**Model 4.3 (Non-ignorable missingness).**

$$\begin{array}{rl} & y_{ij}|p_{i}\overset{i.i.d.}{∼}\text{Bernoulli }(p_{i})\\ & r_{ij}|\pi_{1i},y_{ij}=0\overset{i.i.d.}{∼}\text{Bernoulli }(\pi_{1i}),\\ & r_{ij}|\pi_{2i},y_{ij}=1\overset{i.i.d}{∼}\text{Bernoulli }(\pi_{2i}),\\ & p_{i}|\mu_{p},\tau_{p}\overset{i.i.d.}{∼}\text{Beta}(\mu_{p}\tau_{p},(1-\mu_{p})\tau_{p})\\ & \pi_{1i}|\mu_{\pi_{1}},\tau_{\pi_{1}}\overset{i.i.d.}{∼}\text{Beta}(\mu_{\pi_{1}}\tau_{\pi_{1}},(1-\mu_{\pi_{1}})\tau_{\pi_{1}})\\ \mathrm{and}\;\; & \pi_{2i}|\mu_{\pi_{2}},\tau_{\pi_{2}}\overset{i.i.d.}{∼}\text{Beta}(\mu_{\pi_{2}}\tau_{\pi_{2}},(1-\mu_{\pi_{2}})\tau_{\pi_{2}}) \end{array}$$

For a fully Bayesian analysis, the μ and τ parameters in Model 4.1, Model 4.2 and Model 4.3 need priors. Following the suggestion in Nandram & Choi [34] we give the μ parameters Beta distributions and the τ parameters Gamma distributions.

In §5, we end up using an empirical Bayes approach which uses fixed values of $\mu_{p}$, $\tau_{p}$, $\mu_{\pi_{1}}$, $\tau_{\pi_{1}}$, $\mu_{\pi_{2}}$ and $\tau_{\pi_{2}}$ for the non-ignorable model. We take a moment to introduce this approach here. The methodology is based on the one introduced in Stasny [33]. Briefly, it involves using the maximum-likelihood estimators (MLEs) for $\mu_{p}$, $\tau_{p}$, $\mu_{\pi_{1}}$, $\tau_{\pi_{1}}$, $\mu_{\pi_{2}}$ and $\tau_{\pi_{2}}$ for the marginal distribution of the observed likelihood after integrating out all $p_{i}$, $\pi_{1i}$ and $\pi_{2i}$. More specifically, recalling our ordering convention, we define the number of observed errors for examiner i as

$$E_{i}^{\text{Obs}}=\sum_{j=1}^{R_{i}}y_{ij}.$$

The likelihood function of the observed data is

4.1  f(EObs,R|p,π1,π2)  =∏i=1I(JiRi)(RiEiObs)(piπ2i)EiObs((1−pi)π1i)Ri−EiObs[pi(1−π2i)+(1−pi)(1−π1i)]Ji−Ri,

where $E^{\text{Obs}}={\left(E_{1}^{\text{Obs}},\ldots ,E_{I}^{\text{Obs}}\right)}^{T}$ and $R={\left(R_{1},\ldots ,R_{I}\right)}^{T}$. The probability vectors p, $\pi_{1}$ and $\pi_{2}$ are defined analogously. We obtain the marginal likelihood of the observed data given $\mu_{p}$, $\tau_{p}$, $\mu_{\pi_{1}}$, $\tau_{\pi_{1}}$, $\mu_{\pi_{2}}$ and $\tau_{\pi_{2}}$ by integrating (4.1) with respect to the three beta distributions defined in Model 4.3. Thus, the MLEs are obtained for $\mu_{p}$, $\tau_{p}$, $\mu_{\pi_{1}}$, $\tau_{\pi_{1}}$, $\mu_{\pi_{2}}$ and $\tau_{\pi_{2}}$ via numerical optimization of the following (see Appendix B for more details):

4.2   {B(μpτp,(1−μp)τp)B(μπ1τπ1,(1−μπ1)τπ1)B(μπ2τπ2,(1−μπ2)τπ2)}−I  ×∏i=1I∑z=0Ji−Ri(JiRi)(RiEiObs)(Ji−Riz)B(EiObs+z+μpτp,Ji−EiObs−z+(1−μp)τp)  ×B(Ri−EiObs+μπ1τπ1,Ji−Ri−z+(1−μπ1)τπ1)  ×B(EiObs+μπ2τπ2,z+(1−μπ2)τπ2),

where B(a,b) is the Beta function equal to Γ(a)Γ(b)/Γ(a+b).

### (a) Inference for error rates

We are ultimately interested in the proportion of times that an error will occur. To quantify this, we use the posterior predictive distribution of a variable, $P_{E}$, which is the total number of errors we’d expect to see in a new study divided by the total number of items in the study. The distribution (and definition) of this variable will vary based on the model employed. To describe our approach in more detail, we first introduce one piece of additional notation. We define the number of unobserved errors for examiner i as

$$E_{i}^{\text{Mis}}=\sum_{j=R_{i}+1}^{J_{i}}y_{ij}.$$

Recall, Model 4.1 does not account for the possibility of unobserved errors. Additionally, we intend to use it as the forensic community does: assuming that there are no items beyond those observed. Thus, we consider the study as only consisting of the $R=\sum_{i=1}^{I}R_{i}$ total number of items with a response. We define the proportion of observed errors in the study as

$$P_{E,\text{Naive}}=\frac{\sum_{i=1}^{I^{*}}E_{i}^{\text{Obs}}}{R}.$$

For our inferential goal, we use the posterior predictive distribution of $P_{E}|Y^{\mathrm{obs}}$, where $Y^{\mathrm{obs}}=\{y_{ij}|j=1,\ldots ,R_{i};i=1,\ldots ,I^{*}\}$. We note this distribution is simple to approximate after fitting Model 4.1 using the following observation:

$$E_{i}^{\text{Obs}}∼\text{Binom}(R_{i},p_{i}).$$

Both the ignorable model (Model 4.2) and the non-ignorable model (Model 4.3) account for the possibility of unobserved errors. They inherently assume that the study consisted of all assigned items (regardless of whether there is a response). In other words, we consider $E_{\text{total}}=\sum_{i=1}^{I}=E_{i}^{\text{Obs}}+E_{i}^{\text{Mis}}$ the total number of errors committed in a set of $J=\sum_{i=1}^{I}J_{i}$ items assigned for comparison. Thus for these models, we have the following definition:

$$P_{E,\text{Ignorable}}=P_{E,\text{Non-ignorable}}=\frac{E_{\text{total}}}{J}.$$

We use the posterior predictive distribution of $P_{E}|Y,R$ to derive inference on the error rate. Note, we can do this after fitting the appropriate model despite having not observed the latent variables $E_{i}^{\text{Mis}}$. To do so, we use the fact that for the ignorable model the following holds:

$$E_{i}^{\text{Obs}}∼\text{Binom}(R_{i},p_{i}\pi_{i})$$

and

$$E_{i}^{\text{Mis}}∼\text{Binom}(J_{i}-R_{i},p_{i}(1-\pi_{i})).$$

Similarly, for the non-ignorable model:

$$E_{i}^{\text{Obs}}∼\text{Binom}(R_{i},p_{i}\pi_{2i})$$

and

$$E_{i}^{\text{Mis}}∼\text{Binom}(J_{i}-R_{i},p_{i}(1-\pi_{2i})).$$

## 5. Application

In this section, we consider the black-box study of latent palmar prints presented in Eldridge *et al.* [5]. Using the models introduced in §4, we offer a re-analysis of their data to explore the impact that missing data might be having on error rate estimates. We also explore the impact of treating ‘inconclusives’ as missing responses.

### (a) Latent palmar print black-box data

The palmar print study was designed to assess the accuracy of latent print examiners. There were originally 328 examiners enrolled. However, only 226 returned a response to at least one item, and Eldridge *et al.* [5] based all error rates estimates on responses from these 226 examiners. All participants received 75 items (comparisons) to complete: 22 different source and 53 same source items. As described previously, participants in this study had a multi-stage approach to analysing items. First, examiners assessed the images of the prints for suitability for comparisons. If examiners found an item suitable for comparison, they then could enter a conclusion of Inconclusive, Identification or Exclusion.

**Figure 2.**
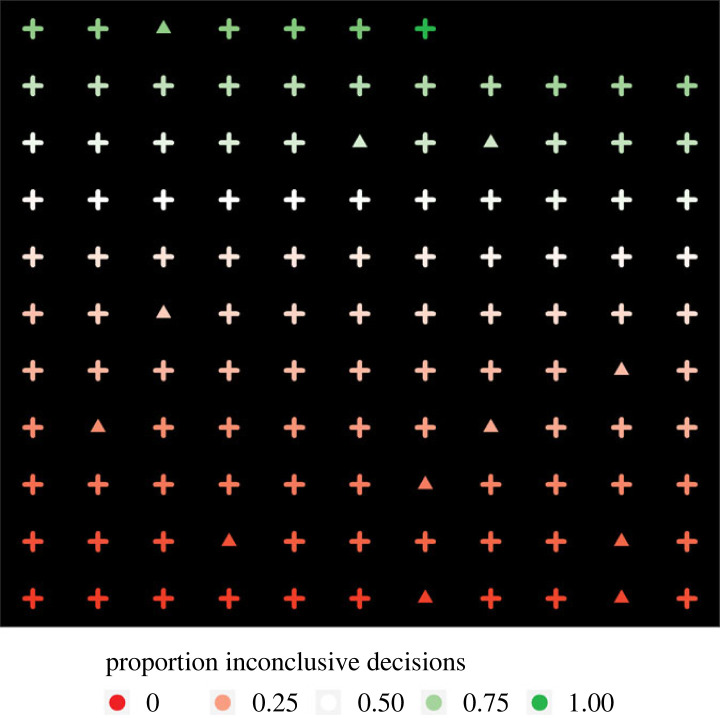
Plot of different source items. The colour references the proportion of examiners who reached a comparison decision of inconclusive. The shape represents whether a false positive occurred on that item (a plus for no and a triangle for yes). All plots were made with the ggplot2 package [36]. (Online version in colour.)

For simplicity, we focus on the inferential goal of estimating the false positive error rate: the proportion of times that examiners conclude ‘Identification’ for different source items. There were 126 unique different source items in the dataset (i.e. 126 items that received at least one response from an examiner). Of these, 117 (93%) were marked inconclusive by at least one examiner. All of these items were analysed by multiple examiners, and the median number of examiners evaluating each of the 117 items was 22. There was seldom agreement across examiners about whether a particular item was inconclusive. In fact, the majority of examiners agreed an item was inconclusive for less than 30% of the 117 items. There did not appear to be a strong relationship between the proportion of examiners who marked an item as inconclusive and occurrence of a false positive on the item. In total, there were 12 false positives. All 12 errors were made on different items. The errors were all made on an item where at least one examiner reached a conclusion of inconclusive. However, only 3 of the 12 errors were made on items where the majority of examiners arrived at a conclusion of inconclusive. In figure 2, we offer a visualization of these observations. In this plot, each shape represents one of the 117 different source items for which at least one examiner reached a decision of inconclusive. The colour represents the proportion of examiners who reached a decision of inconclusive on the item. Red indicates that only a minority of examiners reached a decision of inconclusive, while green indicates the majority of examiners reached a decision of inconclusive. The shapes indicate whether a false positive was made by an examiner on the item: the plus sign indicates no false positive errors occurred and the triangle indicates a false positive occurred.

**Figure 3.**
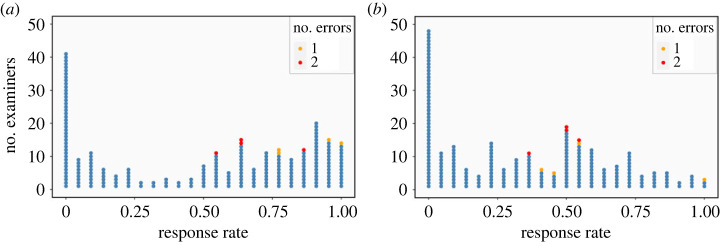
Dotplots of examiners' response rates. All plots were made with the ggplot2 package [36]. (*a*) Response rates by examiner (inconclusive observed) and (*b*) response rates by examiner (inconclusive unobserved). (Online version in colour.)

Each of the 226 participants were assigned 22 different source items. If inconclusive decisions are treated as observed and correct, the non-response rates were quite high. In figure 3*a*, each dot represents one of the 226 examiners. The *x*-axis represents the response rate for the examiner, and the colours of the dots indicate the number of false positives that examiner made in his/her set of comparisons. Forty-one of the examiners failed to reach a single comparison decision, and over 42% of the examiners failed to reach a comparison for the majority of their assigned items. Only 14 participants reached a comparison decision for all of their items. In total, eight examiners made the 12 false positives. There appears to be evidence that as the response rate increases, so too does the likelihood that an examiner will commit a false positive. All of the false positives were committed by examiners who reached a comparison decision on the majority of their decisions. This pattern is supportive of evidence of non-ignorable missingness if examiners were less likely to respond to items on which they might make errors.

When inconclusive decisions were treated as missing, some patterns changed. In figure 3*b*, the non-response rates increase substantially. Now, approximately 67% of the examiners failed to reach a comparison decision on the majority of their assigned items. There is not as clear a trend between response rates and the likelihood of an observed error as there was when inconclusives were treated as correct and observed, although five of the eight examiners who made an error answered 50% or more of their items.

The rates of non-response and inconclusive decisions were quite high. As discussed previously, current analyses ignore both issues. Although our analysis models are not directly comparable to the analysis methods employed in Eldridge *et al.* [5], it can be helpful to have a reference point for comparison. In the study, the authors reported only a point estimate for the false positive error rate in their paper. They considered two point estimates: (i) one where inconclusives were dropped from the analysis and (ii) one where inconclusives were treated as correct decisions. These approaches arrived at point estimates of 0.7% and 0.5%, respectively (although the authors reported the latter as 0.4% with what is perhaps a rounding error). Black-box studies commonly use the Clooper–Pearson estimator for error rates [37]. In table 1, we provide the point estimates and 95% confidence intervals for the false positive error rate using this estimator.

**Table 1.** Clooper–Pearson point estimate and confidence interval for the false positive error rates for the Eldridge *et al.* [5] data for a dataset including inconclusive and a dataset dropping them.

inconclusives	est. (%)	95% conf. int.
obs.	0.5	(0.2%,0.8%)
dropped	0.7	(0.3%,1.2%)

### (b) Hierarchical Bayesian models

In this section, we restrict our attention to the 226 participants who returned at least one response in the study. This is because, as dicussed below, the high rates of missingness began to lead to problems fitting some models when the full 328 were included as part of the study.

To be comparable to the analysis in Eldridge *et al.* [5], we consider the J=4972 different source trials assigned to the 226 participating examiners. For all analyses, we assume that a comparison decision of ‘Identification’ is an error and a decision of ‘Exclusion’ is correct. As described in §2(b), defining what it means to be missing is complicated. In this section, we treat items identified as being of ‘no value’ for comparisons as missing. We do this, in part, out of necessity because the authors of the study have declined to release information regarding items assigned but left unanswered. This precludes us from meaningfully modelling the probability a particular item would have been deemed as no value for the ith examiner. For comparison decisions of inconclusive, we consider two possibilities for a decision of inconclusive: (i) it is treated as ‘correct’ or (ii) it is treated as ‘missing.’

Thus, we have two types of datasets for analysis: (i) a dataset where inconclusives are treated as observed, correct answers and (ii) a dataset where inconclusives are treated as missing. We define Y to be the collection of responses $y_{ij}$, where $y_{ij}$ is 1 if the ith examiner answered ‘Identification’ and 0 if he/she answered ‘Exclusion.’ As noted previously, comparison decisions with a conclusion of ‘Inconclusive’ are treated as either missing or correct (i.e. 0). Similarly, we define R to be the collection of $r_{ij}$’s, where $r_{ij}=1$ if the ith examiner responds to the jth item and 0 otherwise.

We fit Model 4.1, Model 4.2 and Model 4.3 to each type of dataset. The dimensions of the Y and R depend on the model being fit. For Model 4.1, all unobserved responses are dropped from the analysis (i.e. $j=1,\ldots ,R_{i};i=1,\ldots ,I^{*})$. For the Model 4.2 and Model 4.3 models, we do not drop unobserved responses (i.e. j=1,…,22;i=1,…,I)). The implications of treating inconclusives as either correct or missing on these dimensions are summarized in table 2.

**Table 2.** Summary of latent palmar print responses for different source trials. R is the total number of items with a response, J is the total number assigned trials, $I^{*}$ is the total number of examiners with at least one non-missing response and I is the total number of participating examiners reported in Eldridge *et al.* [5].

inconclusives	R	J	$I^{*}$	I
obs.	2560	4972	185	226
missing	1785	4972	178	226

The naive and ignorable models were successfully fit with a fully Bayesian analysis. However, the non-ignorable model was difficult to fit with this approach. The μ and τ parameters showed evidence of only being weakly identifiable and were very slow to mix. We found that only highly informative priors in combination with fixing at least one of $\mu_{\pi_{1}}$ or $\mu_{\pi_{2}}$ allowed these models to be successfully fit. Our explorations with simulated data suggested that these difficulties were likely because of a combination of two factors. The first factor is the exceptionally high rates of non-response. The second factor is the relatively strong positive relationship between response rates and false positive error rates among examiners. Simulated datasets with lower rates of non-response and a weaker relationship between response rates and error rates could be used to successfully fit the non-ignorable models. These factors may very well be present in other black-box studies, so we felt it was important to avoid the use of arbitrary, informative priors in such analyses. Instead, we used an empirical Bayes approach introduced in §4 for the non-ignorable model. In the following discussion, we first describe our efforts with the naive and ignorable models and then discuss the non-ignorable model.

For the naive and ignorable model, the τ parameters were given Γ(2,0.5) priors, where the Gamma distribution is parameterized with shape and rate. All μ parameters were given Beta(1,1) priors. All models were fit with just another Gibbs sampler (JAGs) through the rjags package in the R computing environment [38,39]. Recall, for each model, we considered two datasets: (i) one in which inconclusives are treated as observed (and correct) and (ii) one in which inconclusives are treated as missing. For each model and dataset combination, we ran two Markov Chain Markov Carlo (MCMC) chains. Each chain was run for 15 000 iterations with a burn-in of 5000 for all models, which was sufficient to ensure Monte Carlo standard errors calculated via the batch means proposed in Jones *et al.* [40] were less than 0.01 for all parameters of interest (i.e. all probabilities) (see also, [41]). To further monitor convergence, we employed the multivariate extension of the Gelman–Rubin diagnostic criterion [42]. This statistic was less than 1.1 for all model and data combinations. Full details of the MCMC algorithms employed and convergence criterion, including samples of trace plots are available in Appendix B.

For the non-ignorable model, $\mu_{p}$, $\tau_{p}$, $\mu_{\pi_{1}}$, $\tau_{\pi_{1}}$, $\mu_{\pi_{2}}$ and $\tau_{\pi_{2}}$ were fixed to the MLEs for (4.2) introduced in §4. These values were obtained through numerical optimization, as detailed in Appendix B. For the dataset where inconclusives were treated as observed and correct, this resulted in the following approximate values (rounded here but not in the analysis): $\mu_{p}=0.2$, $\tau_{p}=7.1$, $\mu_{\pi_{1}}=0.6$, $\tau_{\pi_{1}}=0.4$, $\mu_{\pi_{2}}=0.01$ and $\tau_{\pi_{2}}=3.5$. We note that the convergence to very small values of $\mu_{\pi_{2}}$ was found using two different parametrizations of (4.2). It appears to have been driven, in part, by the positive relationship between response and observed error rates. Similarly, when inconclusives were treated as missing, the following approximate values were obtained: $\mu_{p}=0.4$, $\tau_{p}=4.6$, $\mu_{\pi_{1}}=0.64$, $\tau_{\pi_{1}}=0.32$, $\mu_{\pi_{2}}=0.006$ and $\tau_{\pi_{2}}=8.0$. For both datasets, the MLEs for $\mu_{\pi_{1}}$, $\tau_{\pi_{1}}$, $\mu_{\pi_{2}}$ and $\tau_{\pi_{2}}$ tell a similar story. These estimates reflect a prior belief that examiners will likely respond to an item if an error is not made and unlikely to respond to an item if an error is made. However, the MLEs for $\mu_{p}$, $\tau_{p}$ are a bit more different for the two datsets. Effectively, for the dataset where inconclusives are treated as missing, the MLEs for these parameters reflect a prior belief that examiners are more likely to make errors than the respective MLEs for the dataset where inconclusives are treated as observed. After fixing these values, the non-ignorable models were fit with JAGs. Because some of the μ parameters were so small and several τ values were below 1, we used truncated Beta and Gamma priors to avoid numerical problems in the slice samplers used by JAGs. Specifically, the Beta priors were truncated to the interval (0.001,0.999) and the Gamma priors were truncated to the interval (0.001,∞).

As before, two chains were run for each dataset. For the dataset where inconclusives were treated as observed, each chain was ran for 40 000 iterations with a burn-in of 10 000. This was sufficient to ensure that the Monte Carlo standard errors were less than 0.01 for all parameters. The Gelman–Rubin diagnostic criterion was 1.2. For the dataset where inconclusives were treated as missing, each chain was run for 60 0000 iterations with a burn-in of 10 000. This again ensured all Monte Carlo standard errors were less than 0.01. The Gelman–Rubin diagnostic criterion was 1.2 for this model. As before, full details of the MCMC algorithms employed and convergence criterion, including samples of trace plots, are available in Appendix B.

After fitting each model, we approximated the posterior predictive distribution for the relevant $P_{E}$ (as defined in §4(a)). We summarize the mean and the equal-tailed 95% credible interval for these posterior predictive distributions in table 3.

**Table 3.** Summary of the predictive posterior distribution for $P_{E}$

model	inconclusives	$E(P_{E}|Y,R)(\%)$	95% cred.int.
Model 4.1 (naive)	obs.	0.6	(0.2%, 1.5%)
Model 4.1 (naive)	missing	0.8	(0.2%, 1.9%)
Model 4.2 (ignorable)	obs.	0.5	(0.2%, 0.9% )
Model 4.2 (ignorable)	missing	0.6	(0.2%, 1.1% )
Model 4.3 (non-ignorable)	obs.	8.4	(7.2%, 9.7% )
Model 4.3 (non-ignorable)	missing	28.4	(26.3%, 30.5%)

We note that our naive model yields inferences quite similar to those of the Clooper–Pearson estimators summarized in table 1, indicating this model serves well as a proxy for what is currently being done in black-box studies. The 95% credible intervals are wider than the 95% confidence interval, which is not unexpected given we are working with a posterior predictive distribution. Typically, a model which accounts for ignorable missingness will have less uncertainty associated with its estimates than a model that does not account for this missingness. However, we would not expect the point estimates to change much. In other words, for a fixed dataset, we expect the 95% credible intervals for $P_{E}$ associated with Model 4.2 to be narrower than the one associated with Model 4.1. As can be seen in table 3, that trend is observed here.

It is really only for the non-ignorable model that we see significant differences in inference based on whether we treat inconclusives as observed or missing. For both models, the estimated proportion of errors in a study is much higher than for either the naive or ignorable model. This is not particularly surprising given the fact that the MLEs for the hyper-parameters resulted in priors that indicated examiners would be unlikely to respond in the event of an error (i.e. a prior that assumed $\pi_{2i}$’s were centred close to 0) for both datasets. The predicted error rate increased substantially when inconclusives were treated as missing. This is likely due to the combination of the difference in MLEs (i.e. the MLE for $\mu_{p}$ was higher when inconclusives were treated as missing than when they were treated as observed) and the incredibly high non-response rates. The non-response rate when inconclusives are treated as observed is 48.5% (note we are dropping the distinction between unit and item non-response for the moment). This rate increases to 64% when inconclusives are treated as missing, meaning that the majority of comparison decisions are now being imputed. Overall, these findings suggest that the inferences in this black-box study are heavily dependent on the missingness mechanism. If missingness is truly non-ignorable, naively ignoring the missingness could result in gross underestimates of error rates for a study.

**Figure 4.**
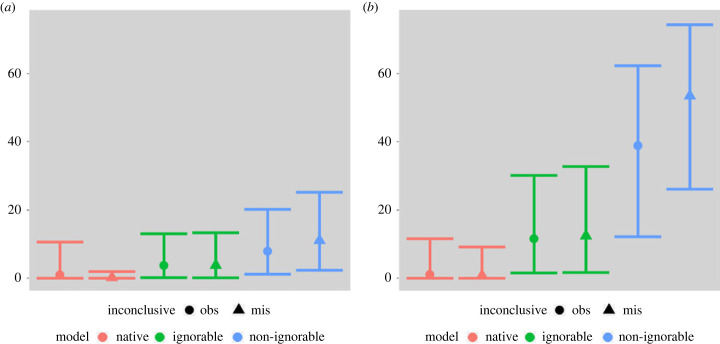
95% credible intervals for the marginal posterior distributions for $p_{i}$s corresponding to Examiner A and Examiner B. All plots were made with the ggplot2 package [36]. (*a*) Probability of error (%) for Examiner A and (*b*) probability of error (%) for Examiner B. (Online version in colour.)

Until now, we have focused on study-level inferential goals. Eldridge *et al.* [5] and other black-box studies have expressed interest in deriving estimates for examiner-specific error rates. Currently, these efforts ignore the missingness. To the extent that examiners are ‘similiar’ (i.e. it makes sense to model examiners error rates as coming for a parent population), Bayesian heiarchical models such as these can help to adjust for missingness for examiner level inferential goals. For the ignorable and non-ignorable models, this could be done with the posterior distributions of the $p_{i}$’s. As an example, we could imagine wanting to derive error rate estimates for two of the error-prone examiners. Let us call the examiner who answered all 22 assigned items, committed one error and had no inconclusive decisions Examiner A (see figure 3) Similarly, let us call the examiner who answered 12 of the assigned items, committed two errors and gave one inconclusive decision Examiner B. We can calculate the 95% credible intervals for both Examiner A and Examiner B’s individual error rates for every model we have considered. In figure 4, we plot these credible intervals for consideration. In this plot, the colours indicate the analysis model. The shapes indicate the posterior means, with circles representing estimates from models where inconclusives were treated as observed and triangles representing estimates from models where inconclusves were treated as missing.

Examiner A had a 0% non-response rate and an empirical error rate of approximately 4.5%. In figure 4*a*, we note that the naive models would have suggested that this examiner’s error rate was lower than that observed, the ignorable models would have suggested this error rate was similar to the observed one, and both non-ignorable models would have suggested higher error rates. When inconclusives were treated as observed, Examiner B had a non-response rate of 45.4% and an empirical error rate of 16.7%. On the other hand, when inconclusive were treated as missing, Examiner B had a non-response rate of 50.0% and an empirical error rate of 18.0%. In figure 4*b*, we note that the naive models would have suggested that this examiner’s error rate was significantly lower than that observed, the ignorable models would have suggested this error rate was slightly smaller than the observed one, and both non-ignorable models would have suggested higher error rates.

## 6. Discussion

Judges and juries in the U.S. criminal justice system are frequently assured that the error rates made by forensic practitioners are negligibly small. These statements were previously made with no empirical evidence to support them. Since the PCAST (2016) report, the results of recent black-box studies are now being used to offer support for these claims. However, the small empirical error rates ignore incredibly high rates of missingness in the data.

In this paper, we have provided an in-depth review of the types of missingness that are currently occurring in black-box studies, and we have highlighted the incredibly complex patterns to this missingness. We observed that with the combination of decisions that items are of ‘no value’ for comparison or that the comparison results in an ‘inconclusive’ decision, current error rates estimations in black-box studies effectively rely on datasets where over 50% of the comparison decisions are unobserved. For example, in the palmar print case study considered in this paper, 7436 different source items were originally assigned to 328 examiners. However, only 226 examiners gave any response. These examiners only reached a comparison decision (if we include inconclusives as observed) for 2560 of them. In any other scientific field, a non-response rate of 65% would preclude any conclusions about a discipline-wide error rate. Yet, in forensic science, researchers not only consistently arrive at such conclusions, but they communicate them to the legal community *without any acknowledgement* of the missingness. U.S. courts are explicitly told that the results obtained in these studies *are* generalizable to the wider population of examiners (e.g. [43]).

There is a wide area of research into methods of handling missing data. However, the data from forensic black-box studies are frequently withheld from independent researchers and the public. In other words, the data necessary to assess the impact of missingness are unavailable to the experts capable of carrying out such an assessment. In this paper, we have proposed a set of models, for either ignorable or non-ignorable missingess, that rely on no auxiliary information. We have illustrated how to fit these models through a fully Bayesian analysis, and, in the case of non-ignorable models, through an empirical Bayes approach. We have also provided a meaningful way of summarizing the impact of such missingness through the use of the posterior predictive distribution of what we called $P_{E}$, the proportion of times an error is expected to occur in a new study with the same number of trials. We found evidence that these models could give some insight into the impact of *item* non-response for the latent print black-box study when inconclusives are treated as observed.

We propose that both the ignorable and non-ignorable models could be fit to existing results from black-box studies to explore the potential impact of item non-response, and the results for each should be presented to courts. These models only require information on response rates and decisions of examiners, ensuring there is no reasonable concern with identifiability of examiners. While the results would still not reflect an estimate of the ‘true’ error rate among examiners, it would present a more honest picture of the potential magnitude of such an error rate given the very high level of missingness in these studies. We emphasize that this suggestion is made in the context of the current state of affairs in the forensic and legal community. This is a world in which (i) tremendously high rates of missingness are observed in black-box studies, (ii) this missingness is ignored completely in subsequent analyses, (iii) judges and juries are told they can rely on these analyses to conclude error rates are very small in forensic disciplines, (iv) researchers are unwilling or unable to explore the patterns of missingness and (v) the data are not released to independent researchers able and willing to conduct such explorations.

Estimates of error rates obtained from the non-ignorable model are very high relative to the other model estimates, in particular when inconclusive decisions are modelled as missing. We believe—although we cannot demonstrate—that incorporating additional information about items and examiners would result in lower error rates than those we obtained. In our analyses, the MLEs of the hyper-parameters in the non-ignorable model that were fixed in the empirical Bayes approach were driven to the boundaries because of the effect of low error rate, the association between observed errors and response rates, and the assumption that all error and response probabilities are generated from a common Beta distribution. Any information that would permit relaxing the reliance on only observed error rates and response rates (e.g. a covariate to help model the error rate probability) might help keep the corresponding hyper-parameters away from the boundaries and would lower estimated error rates.

In future work, particularly with the availability of more auxiliary information, we believe that the SM approach to modelling can be used to better account for missingness. As a first step, unit and item non-response should likely be modelled separately. In this paper, we have blurred the lines in our case study to match the analyses currently being done in black-box studies. However, it is likely, for example, that the error rates of examiners who give no responses may be different from the error rates of examiners who respond to every assigned item. Additionally, inconclusives should likely be modelled more explicitly. In particular, data from the palmar prints study considered in this paper suggest that the relationship between non-response and error rates may be different than the relationship between error rates and the rate at which an examiner arrives at inconclusive decisions. An analysis model that accounts for the possibility of an inconclusive decision for the missing values *and* models, for example, the probability a binary decision would have been an error given that an inconclusive decision was observed on an item, may be more suitable for black-box studies.

## Figures and Tables

**Figure 5.**
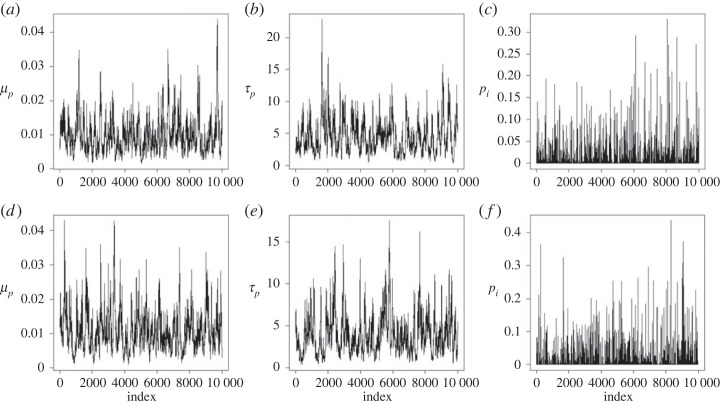
A sampling of trace plots for parameters for naive models. (*a*)–(*c*) The model in which Inconclusives are treated as observed. (*d*)–(*f*) The model in which Inconclusives are treated as missing. (*a*) $\mu_{p}$, (*b*) $\tau_{p}$, (*c*) a $p_{i}$, (*d*) $\mu_{p}$, (*e*) $\tau_{p}$ and (*f*) a $p_{i}$.

**Figure 6.**
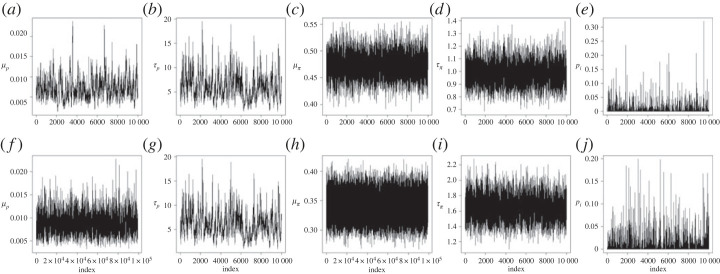
A sampling of trace plots for parameters for ignorable models. (*a*)–(*e*) The model in which Inconclusives are treated as observed. (*f*)–(*j*) The model in which Inconclusives are treated as missing. (*a*) $\mu_{p}$ Inconclusives obs., (*b*) $\tau_{p}$ Inconclusives obs., (*c*) $\mu_{\pi }$ Inconclusives obs., (*d*) $\tau_{\pi }$ Inconclusives obs., (*e*) a $p_{i}$ Inconclusives obs., (*f*) $\mu_{p}$, (*g*) $\tau_{p}$, (*h*) $\mu_{\pi }$, (*i*) $\tau_{\pi }$ and (*j*) a $p_{i}$.

**Figure 7.**
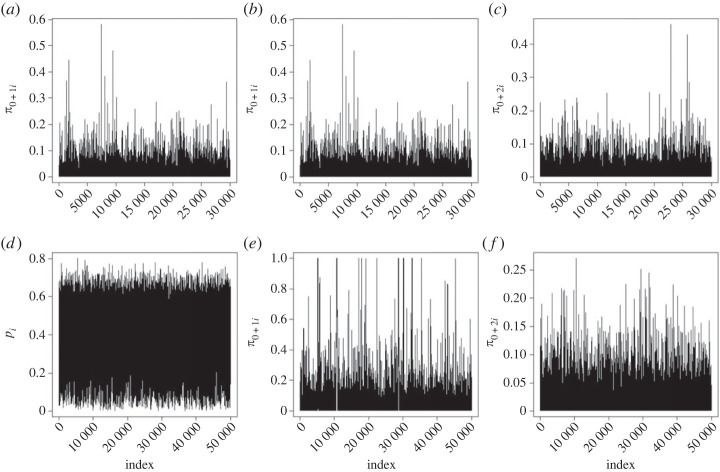
A sampling of trace plots for parameters for ignorable models. (*a*)–(*c*) The model in which Inconclusives are treated as observed. (*d*)–(*f*) The model in which Inconclusives are treated as missing. (*a*) a $p_{i}$, (*b*) a $\pi_{1i}$, (*c*) a $\pi_{2i}$, (*d*) a $p_{i}$, (*e*) a $\pi_{1i}$ and (*f*) a $\pi_{2i}$.

## Data Availability

All code is available with Iowa State University’s DataShare (http://doi.org/10.25380/iastate.22207378) [44]. The data original to the palmar latent print study is available as described in Eldridge *et al.* [5].

## Appendix A. Additional background on black-box studies

**(a) Recent black-box studies by discipline**

— *Bloodstain:* Hicklin *et al.* [7]
— *Digital:* Guttman *et al.* [6]
— *Firearm and toolmark:* Smith *et al.* [12], Smith [11], Monson *et al.* [9] and Chumbley *et al.* [17] (same study)
— *Footwear:* Richetelli *et al.* [10]
— *Handwriting:* Hicklin *et al.* [7]
— *Latent prints:* Ulery *et al.* [18] and Eldridge *et al.* [5]

## Appendix B. MCMC details

All packages were fit using JAGs through the rjags packge in R [38,39,44,45].

**(a) Naive model**

For each of the naive models, two chains were run, each with an adaption interval of 10 000, a burn-in of 5000 and 10 000 saved iterations. When inconclusives were treated as observed, the 95% upperbound for the univariate Gelman–Rubin diagnostic criterion reached a maximum of 1.06 across all parameters [46]. The multivariate extension of the Gelman–Rubin diagnostic criteron was 1.02 [42]. When inconclusives were treated as missing, the 95% upperbound for the univariate Gelman–Rubin diagnostic criterion reached a maximum of 1.09 across all parameters. The multivariate extension of the Gelman–Rubin diagnostic criteron was 1.03. Figure 5 shows a sampling of the traceplots for each model. These traceplots include the parameters with the largest univariate Gelman–Rubin diatnostic criterion for each model.

**(b) Ignorable model**

For each of the ignorable models, two chains were run, each with an adaption interval of 10 000, a burn-in of 5000 and 10 000 saved iterations. When inconclusives were treated as observed, the 95% upper bound for the univariate Gelman–Rubin diagnostic criterion reached a maximum of 1.1 across all parameters. The multivariate extension of the Gelman–Rubin diagnostic criteron was 1.07. When inconclusives were treated as missing, the 95% upperbound for the univariate Gelman–Rubin diagnostic criterion reached a maximum of 1.05 across all parameters. The multivariate extension of the Gelman–Rubin diagnostic criteron was also 1.05. Figure 6 shows a sampling of the trace plots for each model.

**(c) Non-ignorable model**

For each of the two datasets, we first arrived at MLE estimates for the six μ and τ parameters. Briefly, we describe the approach to optimizing (4.2). We can re-write (4.2) as follows:

$$\begin{array}{rl} & \;\prod_{i=1}^{I}\sum_{z=1}^{J_{i}-R_{i}}\exp\{\log\{\Gamma (J_{i}+1)\}-\log\{\Gamma (E_{i}^{\text{Obs}}+1)\}-\log\{\Gamma (R_{i}-E_{i}^{\text{Obs}}+1)\}-\log\{\Gamma (z+1)\}\\ & \;\;-\log\{\Gamma (J_{i}-R_{i}-z+1)\}-\log\{B(\mu_{p}\tau_{p},(1-\mu_{p})\tau_{p})\}-\log\{B(\mu_{\pi_{1}}\tau_{\pi_{1}},(1-\mu_{\pi_{1}})\tau_{\pi_{1}})\}\\ & \;\;-\log\{B(\mu_{\pi_{2}}\tau_{\pi_{2}},(1-\mu_{\pi_{2}})\tau_{\pi_{2}})\}+\log\{B(E_{i}^{\text{Obs}}+z+\mu_{p}\tau_{p},J_{i}-E_{i}^{\text{Obs}}-z+(1-\mu_{p})\tau_{p})\}\\ & \;\;+\log\{B(R_{i}-E_{i}^{\text{Obs}}+\mu_{\pi_{1}}\tau_{\pi_{1}},J_{i}-R_{i}-z+(1-\mu_{\pi_{1}})\tau_{\pi_{1}})\}\\ & \;\;+\log\{(E_{i}^{\text{Obs}}+\mu_{\pi_{2}}\tau_{\pi_{2}},z+(1-\mu_{\pi_{2}})\tau_{\pi_{2}})\}\} \end{array}$$

We used the logarithm of this re-expression in the maximization. Specifically, we used the optim function in R to find the MLEs with the methdology proposed by Byrd *et al.* [47] and lower bounds of 0.001 for all parameters. For the original parameterization above, $\mu_{\pi_{1}}$ converged to 1 very quickly, skipping over potential values for which the log likelihood was higher. Thus, we instead used Beta (α,β) parameterizations for all of the beta components.

To fit each of the non-ignorable models, two chains were run. When inconclusives were treated as observed, each chain had an adaption interval of 10 000 and a burn-in of 10 000. The MCMC was run for 30 000 iterations with a thinning interval of 3. The 95% upper bound for the univariate Gelman–Rubin diagnostic criterion reached a maximum of 1.2 across all parameters. The multivariate extension of the Gelman–Rubin diagnostic criterion was 1.2.

When inconclusives were treated as missing, each chain had an adaption interval of 10 000 and a burn-in of 10 000. The MCMC was then ran for 50,000 iterations with a thinning interval of 5. the 95% upperbound for the univariate Gelman–Rubin diagnostic criterion reached a maximum of 1.4 across all parameters. The multivariate extension of the Gelman–Rubin diagnostic criteron was 1.2. Figure 7 shows a sampling of the trace plots for each model.
